# Iodido(tri-*tert*-butyl­phosphane-κ*P*)gold(I)

**DOI:** 10.1107/S1600536812018569

**Published:** 2012-04-28

**Authors:** Inge Sänger, Hans-Wolfram Lerner, Tanja Sinke, Michael Bolte

**Affiliations:** a Institut für Anorganische und Analytische Chemie, Goethe-Universität Frankfurt, Max-von-Laue-Strasse 7, 60438 Frankfurt am Main, Germany

## Abstract

The Au^I^ atom of the title compound, [AuI(C_12_H_27_P)], shows an almost linear coordination, with a P—Au—I angle of 178.52 (3)° [Au—P = 2.2723 (14) Å and Au—I = 2.5626 (6) Å].

## Related literature

For synthetic background, see: Schödel *et al.* (2006 ▶). For a related compound, [Au(^*t*^Bu_3_P)Cl], see: Schmidbaur *et al.* (1992 ▶). For a description of the Cambridge Structural Database, see: Allen (2002 ▶).
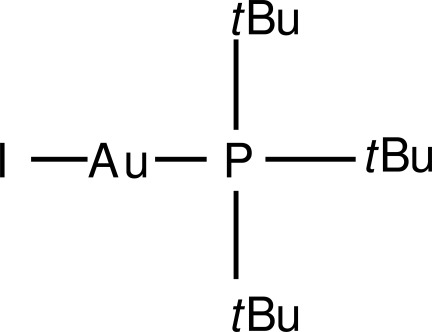


## Experimental

### 

#### Crystal data

[AuI(C_12_H_27_P)]
*M* *_r_* = 526.17
Triclinic, 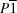

*a* = 7.8198 (11) Å
*b* = 8.9417 (13) Å
*c* = 12.3507 (19) Å
α = 85.325 (12)°
β = 72.840 (12)°
γ = 80.411 (12)°
*V* = 813.1 (2) Å^3^
*Z* = 2
Mo *K*α radiation
μ = 11.02 mm^−1^
*T* = 173 K
0.19 × 0.17 × 0.15 mm

#### Data collection

Stoe IPDS-II two-circle diffractometer
Absorption correction: multi-scan (*MULABS*; Spek, 2009 ▶; Blessing, 1995 ▶) *T*_min_ = 0.229, *T*_max_ = 0.289
16028 measured reflections
3761 independent reflections
3615 reflections with *I* > 2σ(*I*)
*R*_int_ = 0.095

#### Refinement

*R*[*F* ^2^ > 2σ(*F* ^2^)] = 0.038
*wR*(*F* ^2^) = 0.096
*S* = 1.11
3761 reflections
136 parameters
H-atom parameters constrained
Δρ_max_ = 2.30 e Å^−3^
Δρ_min_ = −2.56 e Å^−3^

### 

Data collection: *X-AREA* (Stoe & Cie, 2001 ▶); cell refinement: *X-AREA*; data reduction: *X-AREA*; program(s) used to solve structure: *SHELXS97* (Sheldrick, 2008 ▶); program(s) used to refine structure: *SHELXL97* (Sheldrick, 2008 ▶); molecular graphics: *XP* in *SHELXTL* (Sheldrick, 2008 ▶); software used to prepare material for publication: *SHELXL97*.

## Supplementary Material

Crystal structure: contains datablock(s) I, global. DOI: 10.1107/S1600536812018569/ng5264sup1.cif

Structure factors: contains datablock(s) I. DOI: 10.1107/S1600536812018569/ng5264Isup2.hkl

Additional supplementary materials:  crystallographic information; 3D view; checkCIF report

## Figures and Tables

**Fig. 1.**
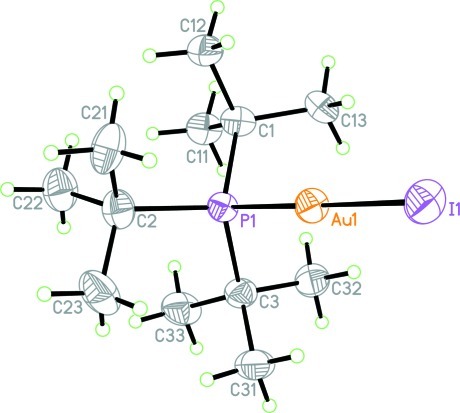
A perspective view of the title compound, showing the atom-numbering scheme. Displacement ellipsoids are drawn at the 50% probability level.

**Fig. 2.**
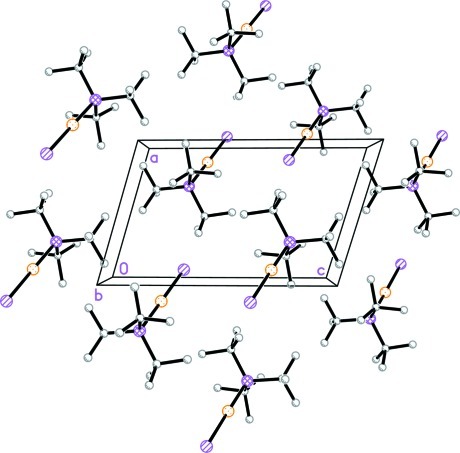
Packing diagram of the title compound with view onto the ac plane. H atoms are omitted.

**Fig. 3.**
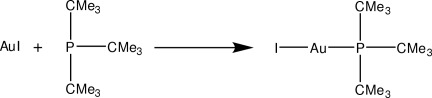
Reaction scheme for the synthesis obtaining the title compound.

## supplementary crystallographic information

### Comment

Recently we have shown that [Au(PF_3_)Cl] could be synthesized when AuCl was treated with PF_3_ in toluene at low temperature (208 K) (Schödel *et al.*, 2006). The solid-state structure of [Au(PF_3_)Cl] reveals attractive interactions between the gold atoms [3.3495 (9) Å]. For closed-shell atoms like Au(I), these interactions can only be rationalized by relativistic effects. In this paper we report the structure of the gold phosphane complex [Au(P*^t^*Bu_3_)I] which we obtained from the reaction of AuI with P*^t^*Bu_3_ at room temperature. In this context it should be noted that Schmidbaur and coworkers had synthesized the related chloro complex [Au(P*^t^*Bu_3_)Cl] from tetrachloroauric acid and *^t^*Bu_3_P (Schmidbaur *et al.*, 1992).

The gold centre of the title compound shows an essentially linear coordination with a P—Au—I angle of 178.52 (3)°. A search in the Cambridge Crystallographic Database (CSD, Version 5.33 of November 2011, plus one update; Allen, 2002) yielded mean values of 2.28 (3) Å for a Au—P bond and of 2.55 (5) Å for a Au—I bond. These values compare well with 2.2723 (14) Å for Au1—P1 and 2.5626 (6) Å for Au1—I1. The crystal packing does not reveal any short intermolecular Au···Au, Au···I nor I···I contact. The shortest values found are: Au1···Au1^i^ 5.8551 (9) Å, Au1···I1^i^ 4.9305 (9) Å, I1···I^i^ 5.2412 (12) Å [symmetry operator (i): -*x* + 2, -*y*, -*z* + 1]. For comparison, the shortest Au···Au contact in [Au(PF_3_)Cl] (Schödel *et al.*, 2006) amounts to 3.3495 (9) Å. It is remarkable that [Au(P*^t^*Bu_3_)Cl] (Schmidbaur *et al.*, 1992) is not isostructural with the title compound and does not show any close Au···Au contact, neither. The shortest Au···Au distance is 6.665 Å.

### Experimental

A mixture of *^t^*Bu_3_P (0.008 g, 0.04 mmol) and AuI (0.012 g, 0.04 mmol) was treated with 3 ml THF. The reaction mixture was stirred for 18 h at room temperature. After filtration single crystals of [Au(*^t^*Bu_3_P)I] were obtained after 10 days at room temperature (yield 53%).

### Refinement

H atoms were refined using a riding model, with C—H = 0.98 Å and with *U*_iso_(H) = 1.5*U*_eq_(C). The highest peak in the final difference density (2.30 e^-^/Å^3^) map is at 0.86 Å from Au1 and the deepest hole (-2.56 e^-^/Å^3^) map is at 0.97 Å from Au1.

### Crystal data

**Table:**

[AuI(C_12_H_27_P)]	*Z* = 2
*M**_r_* = 526.17	*F*(000) = 492
Triclinic, *P*1	*D*_x_ = 2.149 Mg m^−^^3^
Hall symbol: -P 1	Mo *K*α radiation, λ = 0.71073 Å
*a* = 7.8198 (11) Å	Cell parameters from 39755 reflections
*b* = 8.9417 (13) Å	θ = 3.3–28.0°
*c* = 12.3507 (19) Å	µ = 11.02 mm^−^^1^
α = 85.325 (12)°	*T* = 173 K
β = 72.840 (12)°	Block, colourless
γ = 80.411 (12)°	0.19 × 0.17 × 0.15 mm
*V* = 813.1 (2) Å^3^	

### Data collection

**Table:**

Stoe IPDS-II two-circle diffractometer	3761 independent reflections
Radiation source: Genix 3D IµS microfocus X-ray source	3615 reflections with *I* > 2σ(*I*)
Genix 3D multilayer optics monochromator	*R*_int_ = 0.095
ω scans	θ_max_ = 27.7°, θ_min_ = 3.3°
Absorption correction: multi-scan (*MULABS*; Spek, 2009; Blessing, 1995)	*h* = −10→10
*T*_min_ = 0.229, *T*_max_ = 0.289	*k* = −11→11
16028 measured reflections	*l* = −16→15

### Refinement

**Table:**

Refinement on *F*^2^	Primary atom site location: structure-invariant direct methods
Least-squares matrix: full	Secondary atom site location: difference Fourier map
*R*[*F*^2^ > 2σ(*F*^2^)] = 0.038	Hydrogen site location: inferred from neighbouring sites
*wR*(*F*^2^) = 0.096	H-atom parameters constrained
*S* = 1.11	*w* = 1/[σ^2^(*F*_o_^2^) + (0.0492*P*)^2^ + 0.9212*P*] where *P* = (*F*_o_^2^ + 2*F*_c_^2^)/3
3761 reflections	(Δ/σ)_max_ = 0.001
136 parameters	Δρ_max_ = 2.30 e Å^−^^3^
0 restraints	Δρ_min_ = −2.56 e Å^−^^3^

### Special details

**Table:**

Geometry. All e.s.d.'s (except the e.s.d. in the dihedral angle between two l.s. planes) are estimated using the full covariance matrix. The cell e.s.d.'s are taken into account individually in the estimation of e.s.d.'s in distances, angles and torsion angles; correlations between e.s.d.'s in cell parameters are only used when they are defined by crystal symmetry. An approximate (isotropic) treatment of cell e.s.d.'s is used for estimating e.s.d.'s involving l.s. planes.
Refinement. Refinement of *F*^2^ against ALL reflections. The weighted *R*-factor *wR* and goodness of fit *S* are based on *F*^2^, conventional *R*-factors *R* are based on *F*, with *F* set to zero for negative *F*^2^. The threshold expression of *F*^2^ > σ(*F*^2^) is used only for calculating *R*-factors(gt) *etc*. and is not relevant to the choice of reflections for refinement. *R*-factors based on *F*^2^ are statistically about twice as large as those based on *F*, and *R*- factors based on ALL data will be even larger.

### Fractional atomic coordinates and isotropic or equivalent isotropic displacement parameters (Å^2^)

**Table:**

	*x*	*y*	*z*	*U*_iso_*/*U*_eq_	
Au1	0.89137 (2)	0.02882 (2)	0.290901 (16)	0.03077 (9)	
I1	1.10032 (6)	−0.20111 (4)	0.34174 (4)	0.04174 (12)	
P1	0.70393 (18)	0.22883 (15)	0.24297 (11)	0.0274 (3)	
C1	0.4772 (7)	0.2434 (6)	0.3541 (5)	0.0321 (10)	
C2	0.6803 (8)	0.1940 (7)	0.0984 (5)	0.0388 (12)	
C3	0.8073 (7)	0.4076 (6)	0.2365 (5)	0.0353 (11)	
C11	0.3543 (8)	0.3980 (6)	0.3512 (5)	0.0376 (12)	
H11A	0.4167	0.4800	0.3611	0.056*	
H11B	0.2411	0.3986	0.4126	0.056*	
H11C	0.3272	0.4130	0.2781	0.056*	
C12	0.3772 (8)	0.1166 (7)	0.3382 (5)	0.0400 (12)	
H12A	0.4539	0.0183	0.3394	0.060*	
H12B	0.3498	0.1329	0.2653	0.060*	
H12C	0.2642	0.1177	0.3997	0.060*	
C13	0.5073 (8)	0.2136 (6)	0.4724 (5)	0.0365 (12)	
H13A	0.5857	0.1162	0.4739	0.055*	
H13B	0.3905	0.2104	0.5298	0.055*	
H13C	0.5647	0.2951	0.4887	0.055*	
C21	0.6591 (10)	0.0242 (7)	0.0937 (6)	0.0486 (15)	
H21A	0.7595	−0.0414	0.1138	0.073*	
H21B	0.6603	0.0028	0.0169	0.073*	
H21C	0.5442	0.0046	0.1475	0.073*	
C22	0.5239 (10)	0.2946 (8)	0.0668 (6)	0.0487 (14)	
H22A	0.4102	0.2819	0.1247	0.073*	
H22B	0.5182	0.2657	−0.0068	0.073*	
H22C	0.5426	0.4009	0.0621	0.073*	
C23	0.8575 (10)	0.2151 (9)	0.0076 (5)	0.0547 (17)	
H23A	0.9589	0.1506	0.0274	0.082*	
H23B	0.8753	0.3216	0.0034	0.082*	
H23C	0.8516	0.1868	−0.0661	0.082*	
C31	1.0123 (8)	0.3756 (8)	0.1775 (6)	0.0461 (14)	
H31A	1.0668	0.2878	0.2152	0.069*	
H31B	1.0678	0.4645	0.1822	0.069*	
H31C	1.0327	0.3542	0.0978	0.069*	
C32	0.7902 (9)	0.4473 (7)	0.3588 (5)	0.0412 (13)	
H32A	0.6619	0.4691	0.4013	0.062*	
H32B	0.8485	0.5366	0.3573	0.062*	
H32C	0.8492	0.3612	0.3957	0.062*	
C33	0.7229 (9)	0.5455 (7)	0.1780 (6)	0.0475 (15)	
H33A	0.5926	0.5665	0.2157	0.071*	
H33B	0.7427	0.5244	0.0982	0.071*	
H33C	0.7793	0.6339	0.1826	0.071*	

### Atomic displacement parameters (Å^2^)

**Table:**

	*U*^11^	*U*^22^	*U*^33^	*U*^12^	*U*^13^	*U*^23^
Au1	0.03084 (14)	0.02475 (13)	0.03615 (14)	−0.00018 (9)	−0.01127 (9)	0.00028 (9)
I1	0.0430 (2)	0.02648 (19)	0.0591 (3)	0.00003 (15)	−0.02362 (19)	0.00232 (17)
P1	0.0271 (6)	0.0246 (6)	0.0288 (6)	−0.0015 (5)	−0.0069 (5)	−0.0001 (5)
C1	0.028 (2)	0.024 (2)	0.039 (3)	−0.0002 (18)	−0.004 (2)	−0.002 (2)
C2	0.041 (3)	0.040 (3)	0.035 (3)	0.001 (2)	−0.013 (2)	−0.002 (2)
C3	0.033 (3)	0.032 (3)	0.041 (3)	−0.008 (2)	−0.012 (2)	0.007 (2)
C11	0.030 (3)	0.029 (2)	0.048 (3)	0.004 (2)	−0.007 (2)	0.000 (2)
C12	0.041 (3)	0.038 (3)	0.043 (3)	−0.013 (2)	−0.013 (2)	0.004 (2)
C13	0.044 (3)	0.031 (3)	0.031 (3)	−0.006 (2)	−0.006 (2)	0.003 (2)
C21	0.057 (4)	0.047 (3)	0.049 (3)	0.004 (3)	−0.029 (3)	−0.018 (3)
C22	0.051 (4)	0.049 (3)	0.046 (3)	0.004 (3)	−0.020 (3)	0.002 (3)
C23	0.056 (4)	0.066 (4)	0.031 (3)	−0.001 (3)	0.000 (3)	−0.001 (3)
C31	0.033 (3)	0.051 (3)	0.053 (4)	−0.013 (3)	−0.010 (3)	0.012 (3)
C32	0.051 (3)	0.033 (3)	0.046 (3)	−0.014 (2)	−0.018 (3)	−0.002 (2)
C33	0.045 (3)	0.033 (3)	0.060 (4)	−0.005 (2)	−0.013 (3)	0.014 (3)

### Geometric parameters (Å, º)

**Table:**

Au1—P1	2.2723 (14)	C13—H13B	0.9800
Au1—I1	2.5626 (6)	C13—H13C	0.9800
P1—C1	1.887 (5)	C21—H21A	0.9800
P1—C3	1.894 (5)	C21—H21B	0.9800
P1—C2	1.904 (6)	C21—H21C	0.9800
C1—C12	1.535 (7)	C22—H22A	0.9800
C1—C13	1.544 (8)	C22—H22B	0.9800
C1—C11	1.551 (7)	C22—H22C	0.9800
C2—C22	1.521 (9)	C23—H23A	0.9800
C2—C23	1.533 (9)	C23—H23B	0.9800
C2—C21	1.562 (9)	C23—H23C	0.9800
C3—C33	1.531 (8)	C31—H31A	0.9800
C3—C31	1.540 (8)	C31—H31B	0.9800
C3—C32	1.543 (8)	C31—H31C	0.9800
C11—H11A	0.9800	C32—H32A	0.9800
C11—H11B	0.9800	C32—H32B	0.9800
C11—H11C	0.9800	C32—H32C	0.9800
C12—H12A	0.9800	C33—H33A	0.9800
C12—H12B	0.9800	C33—H33B	0.9800
C12—H12C	0.9800	C33—H33C	0.9800
C13—H13A	0.9800		
			
P1—Au1—I1	178.52 (3)	C1—C13—H13C	109.5
C1—P1—C3	110.4 (2)	H13A—C13—H13C	109.5
C1—P1—C2	110.7 (3)	H13B—C13—H13C	109.5
C3—P1—C2	110.2 (3)	C2—C21—H21A	109.5
C1—P1—Au1	108.63 (17)	C2—C21—H21B	109.5
C3—P1—Au1	108.65 (18)	H21A—C21—H21B	109.5
C2—P1—Au1	108.20 (19)	C2—C21—H21C	109.5
C12—C1—C13	106.2 (4)	H21A—C21—H21C	109.5
C12—C1—C11	108.7 (5)	H21B—C21—H21C	109.5
C13—C1—C11	109.6 (4)	C2—C22—H22A	109.5
C12—C1—P1	109.0 (4)	C2—C22—H22B	109.5
C13—C1—P1	109.1 (4)	H22A—C22—H22B	109.5
C11—C1—P1	114.0 (4)	C2—C22—H22C	109.5
C22—C2—C23	109.0 (5)	H22A—C22—H22C	109.5
C22—C2—C21	109.0 (5)	H22B—C22—H22C	109.5
C23—C2—C21	105.5 (6)	C2—C23—H23A	109.5
C22—C2—P1	115.0 (4)	C2—C23—H23B	109.5
C23—C2—P1	108.8 (4)	H23A—C23—H23B	109.5
C21—C2—P1	109.1 (4)	C2—C23—H23C	109.5
C33—C3—C31	109.3 (5)	H23A—C23—H23C	109.5
C33—C3—C32	109.0 (5)	H23B—C23—H23C	109.5
C31—C3—C32	105.3 (5)	C3—C31—H31A	109.5
C33—C3—P1	115.0 (4)	C3—C31—H31B	109.5
C31—C3—P1	109.6 (4)	H31A—C31—H31B	109.5
C32—C3—P1	108.1 (4)	C3—C31—H31C	109.5
C1—C11—H11A	109.5	H31A—C31—H31C	109.5
C1—C11—H11B	109.5	H31B—C31—H31C	109.5
H11A—C11—H11B	109.5	C3—C32—H32A	109.5
C1—C11—H11C	109.5	C3—C32—H32B	109.5
H11A—C11—H11C	109.5	H32A—C32—H32B	109.5
H11B—C11—H11C	109.5	C3—C32—H32C	109.5
C1—C12—H12A	109.5	H32A—C32—H32C	109.5
C1—C12—H12B	109.5	H32B—C32—H32C	109.5
H12A—C12—H12B	109.5	C3—C33—H33A	109.5
C1—C12—H12C	109.5	C3—C33—H33B	109.5
H12A—C12—H12C	109.5	H33A—C33—H33B	109.5
H12B—C12—H12C	109.5	C3—C33—H33C	109.5
C1—C13—H13A	109.5	H33A—C33—H33C	109.5
C1—C13—H13B	109.5	H33B—C33—H33C	109.5
H13A—C13—H13B	109.5		
			
C3—P1—C1—C12	166.5 (4)	Au1—P1—C2—C23	−72.5 (5)
C2—P1—C1—C12	44.2 (5)	C1—P1—C2—C21	−76.8 (5)
Au1—P1—C1—C12	−74.4 (4)	C3—P1—C2—C21	160.8 (4)
C3—P1—C1—C13	−77.9 (4)	Au1—P1—C2—C21	42.1 (4)
C2—P1—C1—C13	159.8 (3)	C1—P1—C3—C33	−75.2 (5)
Au1—P1—C1—C13	41.1 (4)	C2—P1—C3—C33	47.4 (5)
C3—P1—C1—C11	44.9 (5)	Au1—P1—C3—C33	165.8 (4)
C2—P1—C1—C11	−77.4 (4)	C1—P1—C3—C31	161.1 (4)
Au1—P1—C1—C11	163.9 (4)	C2—P1—C3—C31	−76.3 (5)
C1—P1—C2—C22	46.0 (5)	Au1—P1—C3—C31	42.1 (4)
C3—P1—C2—C22	−76.4 (5)	C1—P1—C3—C32	46.8 (5)
Au1—P1—C2—C22	164.9 (4)	C2—P1—C3—C32	169.4 (4)
C1—P1—C2—C23	168.6 (4)	Au1—P1—C3—C32	−72.2 (4)
C3—P1—C2—C23	46.2 (5)		
